# Monitoring the Biodegradation Progress of Naphthenic Acids in the Presence of *Spirulina platensis* Algae

**DOI:** 10.3390/toxics13050368

**Published:** 2025-05-01

**Authors:** Catalina Gabriela Gheorghe, Cristina Maria Dusescu-Vasile, Daniela Roxana Popovici, Dorin Bombos, Raluca Elena Dragomir, Floricel Maricel Dima, Marian Bajan, Gabriel Vasilievici

**Affiliations:** 1 Faculty of Petroleum Refining and Petrochemistry, Petroleum—Gas University of Ploiesti, 39 Bvd. Bucuresti, 100520 Ploiesti, Romania; catalina.gheorghe@upg-ploiesti.ro (C.G.G.); dana_p@upg-ploiesti.ro (D.R.P.); marian.bajan@upg-ploiesti.ro (M.B.); 2 Institute for Research and Development in Aquatic Ecology, Fishing, and Aquaculture, 54 Portului Street, 800211 Galati, Romania; 3 National Institute for Research Development for Chemistry and Petrochemistry-ICECHIM-București, 202 Spl. Independenței, 060021 Bucharest, Romania; gvasilievici@icechim.ro

**Keywords:** microorganisms, bioremediation, pollutants, biodegradation, ECb50

## Abstract

The experiments in this study investigated the toxicity of naphthenic acids (NAs) on the algal culture *Spirulina platensis*. The tests monitored the progression of the algal suspension in media contaminated with various concentrations of naphthenic acids. The evolution of the algal culture during the metabolism of NAs was investigated. The monitoring also included the determination of the values of some parameters during the biodegradation process (pH, conductivity, cell viability, dissolved oxygen). Optical density measurements (OD_600_) were used to quantify the growth of *Spirulina platensis*, alongside the determination of the sedimentation index (IS). Cell viability was assessed microscopically using TEM and optical microscopy. The results facilitated the estimation of the percentage of cell growth inhibition and the inhibitory concentration value, determined by estimating ECb50 (concentration of NAs corresponding to 50% inhibition). The chemical quantification of naphthenic acids in the samples analyzed was performed by calculating the acidity value (AV) and characterizing the naphthenic acids through FTIR analysis. The graphical representation of ECb50 was established by extrapolating to a concentration of 110 mg/mL of naphthenic acids. We have demonstrated that pollution caused by NAs can be mitigated by the algae *Spirulina platensis*, which can metabolize these compounds and thus biodegrade them.

## 1. Introduction

The chemical composition of crude oil primarily consists of hydrocarbons, including naphthenes, paraffins, and aromatic hydrocarbons, which make up around 90% of its content. Additionally, over 10% of the crude oil comprises polar compounds that contain heteroatoms such as nitrogen, oxygen, and sulfur, along with metal atoms. Despite their relatively small proportion, approximately 20,000 polar organic compounds with varying elemental compositions (C_c_H_h_N_n_O_o_S_s_) have been identified in crude oil. These polar compounds can pose challenges during the processes of oil production, refining and storage.

Naphthenic acids (NAs) are present in oil sands process-affected water (OSPW), refinery process water, petroleum products, and wastewater. Typical concentrations of NAs range from 40 to 120 mg/L in OSPW, 0.9 to 3.6 mg/g in heavy crude oil, 4.2 to 40.4 mg/L in refinery desalination brine, and 4.5 to 16.6 mg/L in refinery wastewater treatment effluent. The combination of advanced oxidation processes and microbial biodegradation produces a high-quality effluent with significantly reduced NAs concentrations. However, complex naphthenic acids, which are characterized by multiple rings, aliphatic chains with alkyl substitutions, branched structures, and high molecular weights, are challenging to treat and tend to persist in the environment [1,2,3]. In refineries, naphthenic acids are extracted from the kerosene and diesel distillate fractions to improve the quality and storage properties of the final products.

While naphthenic acids may be undesirable in certain contexts, they can be effectively used after undergoing tartarization to produce metallic soaps, which serve as agents to prevent wood degradation. They can also be transformed into amine derivatives that act as corrosion inhibitors in the tire industry. To address the challenge of removing persistent naphthenic acids, advanced oxidation processes (AOPs), such as ozonation or photocatalysis using UV radiation, are proposed. There are two recognized types of photolysis: direct photolysis, in which organic compounds absorb photons directly, and indirect photolysis, where photosensitizers in water absorb light energy to generate reactive radicals that facilitate the photodegradation of naphthenic acids [4].

An alternative approach for removing naphthenic acids from water involves the adsorption of these acids onto biochar produced from agricultural and wood residues [5]. During bitumen extraction processes, a significant amount of oil sands process-affected water (OSPW) is generated, which contains naphthenic acids. These contaminants can lead to acute toxicity in various organisms, manifesting as narcosis, endocrine disruption, immunotoxicity, and potential carcinogenic effects [6,7].

Some naphthenic acids (NAs) exhibit toxicity towards various aquatic organisms. To mitigate their effects, microalgae have been investigated for their capability to biodegrade NAs [8,9]. The presence of microalgae in waters contaminated with NAs suggests a level of tolerance and detoxification ability. Utilizing microalgae for the removal of NAs presents several additional benefits, such as producing recycled biomass, which serves as a renewable raw material for biofuel production [10]. Various strains of microalgae have been tested for their tolerance to NAs, and many species have proven effective in wastewater treatment systems. Rhamnolipids, part of the glycolipid class, consist of rhamnose combined with fatty acids. These compounds enhance the bacterial metabolism of diverse substances by emulsification and provide protection to certain bacteria [11]. Rhamnolipids reduce surface tension and function as surfactants, promoting bacterial dispersion within environments. As surfactants, they can emulsify substances, thus facilitating access for microorganisms and aiding in their metabolic processes. This characteristic has been leveraged in the bioremediation of petrochemical contaminants. Additionally, rhamnolipids enable certain bacteria to thrive in environments polluted with petrochemical waste. For instance, *Pseudomonas aeruginosa* has frequently been isolated from petrochemical products such as oil, gasoline, and diesel fuel. Research has explored various factors influencing the biodegradation rate, the microbial metabolism of petroleum hydrocarbon pollutants, the degradation pathways of these pollutants, and different bioremediation technologies [11].

Research has identified various bacterial species capable of biodegrading polycyclic aromatic hydrocarbons (PAHs) from petroleum waste, including *Bacillus pumilus, Bacillus subtilis*, *Micrococcus luteus*, *Alcaligenes faecalis*, and *Enterobacter* sp. These microorganisms can effectively degrade compounds such as naphthalene, phenanthrene, fluoranthene, and pyrene. PAHs are classified as highly toxic and persistent due to their low solubility in water, thus rendering microbial degradation a promising solution to the environmental challenges associated with these pollutants. Fractions encompassing n-alkanes exhibit a higher rate of biodegradation, whereas saturated fractions containing branched alkanes are more resistant to degradation. The degradation potential tends to diminish as the number of aromatic or alicyclic rings within a molecule increases [12,13,14,15,16,17]. The bioremediation of aquatic environments and soils contaminated with PAHs relies on the ability of microorganisms to synthesize carbon from these substrates, thus converting them into non-toxic end-products such as water (H_2_O) and carbon dioxide (CO_2_). Microorganisms have the capacity to break specific chemical bonds, including C-C and C=C bonds, as well as to catalyze hydrolysis, decarboxylation, and oxidation reactions. Metabolic processes occur actively between bacteria and nutrient substrates that are fundamentally altered by bacterial enzymes through intricate biochemical reactions. Therefore, any substrate that microorganisms encounter will significantly influence the activity of enzymes targeting its chemical components. Through biological oxidation, microorganisms harness cellular energy released during oxidation processes with the assistance of molecular oxygen. Some microorganisms are specialized in degrading aliphatic compounds, while others are capable of breaking down monoaromatic or polyaromatic substances, as well as resins. Oil pollutants are biodegraded by various bacterial genera, including *Achromobacter*, *Acinetobacter*, *Arthrobacter*, *Azoarcus*, *Brevibacterium*, *Cellulomonas*, *Corynebacterium*, *Flavobacterium*, *Marinobacter*, *Micrococcus*, *Nocardia*, *Ochrobactrum*, *Pseudomonas*, *Stenotrophomonas*, and *Vibrio*. Additionally, fungi from the genera *Aspergillus*, *Amorphoteca*, *Fusarium*, *Graphium*, *Neosartoria*, *Paecilomyces*, *Penicillium*, *Sporobolomyces*, and *Talaromyces*, as well as yeasts from the genera *Candida*, *Pichia*, *Pseudozyma*, *Rhodotorula,* and *Yarrowia*, which also contribute significantly to bioremediation efforts [18,19,20,21].

Naphthenic acids play a significant role in the cosmetic industry and the production of detergents. Additionally, they are generated during extraction processes, where they enhance specific properties of medium petroleum fractions. However, their release into industrial wastewater can lead to contamination, negatively impacting both aquatic and terrestrial ecosystems. As a result, it is essential to neutralize or eliminate these acids. This can be accomplished through traditional industrial methods, as well as various alternative approaches, such as the one explored in this study using microbiological cultures. In our research, we examined the biodegradation of naphthenic acids in the presence of *Spirulina platensis* by assessing the variation in several parameters, including pH, conductivity, cell viability, and oxygen demands.

## 2. Materials and Methods

### 2.1. Preparation of Biological Medium with Spirulina platensis

The biological material used in the experiments was the microalgae *Spirulina platensis*, which was cultivated in Erlenmeyer flasks containing 1 L of Zarrouk medium (NaHCO_3_18 g, NaNO_3_ 2.5 g, K_2_HPO_4_ 0.5 g, K_2_SO_4_ 1.0 g, FeSO_4_ · 7H_2_O 0.01 g, Na_2_EDTA·2H_2_O0.08 g, NaCl 1.0 g, CaCl_2_ · 2H_2_O 0.04 g, MgSO_4_ · 7H_2_O 0.2 g and 1 mL micronutrient solution (containing: H_3_BO_3_ 2.8 g/L; MnCl_2_·4H2O 1.8 g/L CuSO_4_·5H_2_O 0.08 g/L; ZnSO_4_·7H_2_O 0.2 g/L; Na_2_MoO_4_·2H_2_O 0.4 g/L). For the Zarrouk medium, pH was adjusted to 9.5 and was sterilized by autoclaving for 15 min at 121 °C. All the chemicals used throughout the experimental study were of reagent grade (Sigma-Aldrich, St. Louis, MI, USA). An analytical balance OHAUS model AX224M was used for weighing the chemicals.

The monitoring of cell viability during the experiment was carried out using a Celestron Microscope, model 4434. Erlenmeyer flasks were placed in a laboratory shaker (Orbital Multi-Shaker) at 100 rpm, for an optimal aeration at 35 °C ± 1 °C. For the laboratory tests, a multi-parameter WTW Inolab MULTI 9630 IDS was used with three galvanically isolated measuring channels: pH, oxygen, and conductivity measurement The algal cell culture was obtained from the Culture Collection of Petroleum-Gas University of Ploiesti. The mixture of naphthenic acids utilized in this study was derived from a heavy asphaltic crude oil from Romania, specifically Suplacu crude oil, which is recognized for its high concentration of carboxylic acids (1.6% by weight, according to Corinne Whitby, 2010) [22].

### 2.2. Experimental Set-Up

In order to establish the toxic effects of naphthenic acids on the strains grown in the exponential phase, it was necessary to study the growth rate of microorganisms in contact with the naphthenic acids (NAs) analyzed at certain concentrations. For this purpose, five identical series of bioreactors were prepared: Series A, B, C, D and E. Each series consists of seven test Erlenmeyer flasks (bioreactors) labeled as follows: PA1-PA7 for Series A, PB1-PB7 for Series B, PC1-PC7 for Series C, PD1-PD7 for Series D, and PE1-PE7 for Series E. In each bioreactor, a specific quantity of naphthenic acids was weighed gravimetrically in the following amounts (from bioreactor 1 to bioreactor 7 in each series): 0.2 g, 0.4 g, 0.6 g, 0.8 g, 1 g, 1.2 g, and 1.4 g. The concentrations were chosen arbitrarily, with the range of concentration variation being wide to cover common values in cases of accidental spills.

Each series included a reference bioreactor, designated as a blank, corresponding to its series: PAb, PBb, PCb, PDb, and PEb, which did not contain the toxic substance naphthenic acid. In all bioreactors, additional components were added the aliquot volume of inoculum of 10 mL *Spirulina platensis* suspension amounted to 10^4^ cells/mL in the exponential growth phase. In this way, the following concentrations were obtained for each series: 20 mg/mL, 40 mg/mL, 60 mg/mL, 80 mg/mL, 100 mg/mL, 120 mg/mL, and 140 mg/mL. Throughout the testing, the bioreactors were kept in suspension by mechanical stirring in an orbital shaker to improve gas exchange to optimize contact with toxic substance and reduce pH variation in the test solutions. Series A was analyzed after 5 h of incubation, series B after 24 h of incubation, series C after 48 h, series D after 72 h, and series E after 7 days (168 h).

After the incubation period, each bioreactor was evaluated to track the progression of the algal suspension and the concentration of NAs. To achieve this, a series of measurements were conducted, including pH, conductivity, dissolved oxygen (DO) content, and determination of the acidity value (AV) of naphthenic acids (NAs). Additionally, for biological examinations, optical density (OD) readings were conducted and microscopic examinations of algal cells exposed to various concentrations of naphthenic acids were performed, using both optical microscopy and transmission electron microscopy (TEM), for detailed visualization of the cells.

### 2.3. pH, Conductivity, and Dissolved Oxygen Content (DO) Measurement of Spirulina platensis

The pH values, conductivity, and dissolved oxygen content (DO) of bioreactors before initiating the extraction of naphthenic acids were measured with three measurement channels for the three parameters. The biological oxygen demand (BOD) from the analyzed samples was quantified using Equation (1) as follows: Series A was analyzed after 5 h of incubation, Series B after 24 h of incubation, Series C after 48 h, Series D after 72 h, and Series E after 7 days:

BOD = DO_ti_ − DO_tn_, mg/L (1)

where DO_ti_ = the amount of oxygen present in the sample at the initial time of collection and DO_tn_ is the amount of oxygen present in the sample after ten days.

### 2.4. Optical Density Measurement (OD_600_), Quantification of Sedimentation Index (SI), and Microscopic Evaluation of Viability of Spirulina platensis

Cell growth in the culture of microalgae *Spirulina platensis* was assessed spectrophotometrically by measuring the optical density (OD) at a wavelength of 600 nm (OD_600_). For this, an aliquot volume of 1 mL of cell suspension was taken from each bioreactors and was read using a UV–Vis spectrophotometer, model T85+, PG Instruments. To quantify the sedimentation index (IS), the optical density (OD) at a wavelength of 600 nm was measured immediately following sample collection from the bioreactor, as well as after a 10 min resting period. The sedimentation index was calculated using Equation (2). The viability of the algal cells was assessed through microscopic visualization and quantification within the visual field using a Thoma cell counting chamber.

IS = (DO_ti_ −DO_t (10 min)_/DO_ti_) × 100% (2)

where DO_ti_—initial optical density measured immediately after harvesting, and DO_t (10min)_—optical density measured after 10 min of rest.

### 2.5. Inhibition Ratio (IR) and Estimation of CE50

The inhibition ratio (IR) was analyzed by the enumeration of cell numbers every 5 h, 24 h, 48 h, 72 h, and 168 h under an optical microscope (×400). Equation (3) was utilized to determine the percentage of cell growth inhibition (% IR) at every concentration of NA under examination [23,24,25].

IR = Cc − Ct/Ct × 100%(3)

where Cc—cell number density of control culture (cells/mL), and Ct—cell number density of samples with specific NAs concentrations, (cells/mL),

Following the measurement of cell viability, Equation (4) was used to calculate the average specific growth rate (μ), which is an expression referring to the logarithmic increase in biomass throughout the exposure period:

μ = (lnN_n_ − lnN_0_)/(t_0_ − t_n_), day^−1^ (4)

where t_0_ = is the time at the start of the test where the values are represented by measurements performed at 168 ore (t_n_); N_0_ = the number of cells/mL measured at the t_0_; N_n_ = the number of cells/mL measured at t_n_.

By determining the ECb50, the concentration of the test substance that causes a 50% reduction in the analyzed biomass is established. The ECb50 value represents the concentration of NAs that corresponds to a 50% inhibition, and through this quantification, it was possible to establish the toxicity of NAs on the cell development of the algae, thus inducing conclusions for establishing their harmful potential [26,27].

### 2.6. Characterization of the NAs Fraction

#### 2.6.1. Determination of the Acidity Value (AV) of Naphthenic Acids (NAs)

Following the incubation period for each series and after measuring the chemical and biological parameters, the contents of each test tube containing NAs were treated with 20 mL of petroleum ether [14,26,28]. The mixture was homogenized and subsequently transferred to a separatory funnel. The bioreactor was then rinsed twice with 15 mL of a 1:1 (*v*/*v*) alcohol–benzene solvent, and this solution was also transferred to the separatory funnel [23,24]. The funnel was shaken for 2 min and allowed to stand for 5 min to facilitate phase separation. The alcoholic extract was then collected in an Erlenmeyer flask and titrated with 0.1 N KOH using an alkali blue indicator until the indicator changed color from red to blue. The calculation method for AV is presented in Equation (5) [29]

AV= 5.611 × V × f/G, mg KOH/g(5)

where 5.611—the amount of KOH (mg), corresponding to 1 mL of 0.1 N KOH solution, V = volume of 0.1 N KOH solution consumed during titration (mL), F = factor of KOH 0.1 N solution, and G = initial mass of naphthenic acids in the bioreactor (g).

#### 2.6.2. Determination of Unsaponifiable Matter Content

To determine the content of unsaponifiable compounds, 5 g of the NAs sample were mixed with 50 milliliters of a 0.5 N potassium hydroxide solution. The mixture was boiled for one hour. After the saponification reaction was complete, the sample was cooled, and the unsaponifiable compounds were extracted using 30 milliliters of petroleum ether in two successive stages. The petroleum ether in the extract was then recovered through evaporation.

#### 2.6.3. Composition Determination by GC-MS

For the chromatographic analysis a GC/MS Triple Quad Agilent Technology 7890A equipment was used, with NIST library compounds identification. The GC method involves a column HP5-MS 30 m × 250 μm × 0.25 μm, oven program: 80 °C up to 310 °C. The carrier gas: He, flow = 1 mL/min. Injector temp.: 280 °C, injection volume: 0.1 μL, split mode 1:20. For the MS method, a QQQ collision cell was used; quench flow gas (He) = 2.2 mL/min; collision flow gas (N2) = 1.5 mL/min; type of source: EI; electron energy: 70 eV; source temp.: 260 °C; aux temp2: 320 °C; scan segment: 10–600; type of chromatogram: TIC.

#### 2.6.4. The FTIR Spectroscopy Analysis

For the qualitative analysis of the NAs sample used in our experiments, Fourier transform infrared (FTIR) spectroscopy was used to identify the functional groups present in the structure. The equipment used was the Shimadzu IRAffinity-1S spectrophotometer (Kyoto, Japan), equipped with the GladiATR-10 accessory. Measurements were conducted over a wavelength range of 380 to 2000 cm^−1^, with a spectral resolution of 4 cm^−1^.

## 3. Results and Discussion

### 3.1. NAs Fraction Characterization

**Figure 1:**
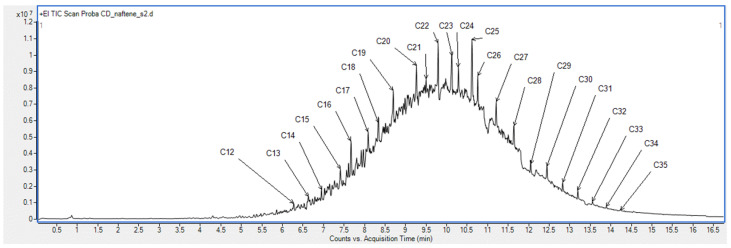
The chromatogram of the NAs sample.

Following the experimental determinations carried out, the acidity value of the NAs fraction was AV = 258.1 mg KOH/g. For this sample, the content of unsaponifiables was determined, with the value obtained being 19.78% wgt. The chromatogram of the NAs sample is shown in Figure 1.

GC-MS analysis reveals the complex composition of the acid sample. The substantial number of components present in the NAs sample—encompassing both carboxylic acids and, notably, aliphatic and aromatic hydrocarbons—amounts to hundreds or even thousands, which is characteristic of heavy petroleum products such as diesel and vacuum distillates. This abundance leads to peak overlap, making it challenging to identify individual components. Consequently, the chromatogram predominantly facilitates the identification of NAs fractions (C12–C35), which are present in higher concentrations than the majority of other compounds, particularly hydrocarbons. For this reason, the error associated with the quantitative determination of NAs from these mixtures is considerable, and the GC-MS method did not provide additional detailed insights into the composition and structure of these compounds [22,30].

### 3.2. FTIR Analysis of NAs Samples

**Figure 2:**
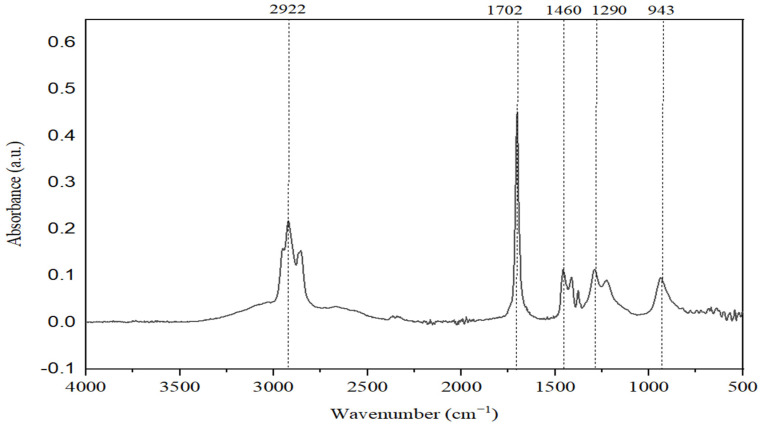
FTIR spectrum of NAs.

To address the GC-MS limitation, we turned to FTIR analysis for further insight into the classes of chemical compounds present—specifically, naphthenic acids or hydrocarbons analytically identified as unsaponifiable. The NAs sample used in our experiments was characterized by FTIR spectroscopy, in the region of 4000–400 cm^−1^. FTIR spectra have distinct bands, and were assigned a range of vibrationally active chemical groups (Figure 2) [31,32].

The spectrum analysis reveals that absorption bands at 3549 cm^−1^ can be observed, indicating the presence of -OH or C-H groups typical of alcohols (ν_OH_ = 3332–3549 cm^−1^) or terminal alkyne (ν_CH_ ≈ 3332 cm^−1^).

The absorption bands in the range of νCH = 2850–2960 cm^−1^ are attributed to the valence vibrations of C-H bonds found in alkanes, while those in the range of νCH = 3000–3100 cm^−1^ correspond to the valence vibrations of aromatic rings. The peaks observed indicate broad absorption bands, which suggest the presence of carboxylic acid compounds, characterized by their molecules being strongly associated through hydrogen bonds (νO-H = 2484–3300 cm^−1^). In the range of 2600–1900 cm^−1^, there are typically very few absorption bands, with any recorded likely related to the valence vibrations of triple bonds. Intense absorptions appear in the region of νC=O = 1680–1725 cm^−1^ due to the valence vibrations of the heterogeneous C=O double bond, a characteristic of carboxylic acids and bands associated with aromatic hydrocarbons in the range of νC=C = 1458–1600 cm^−1^.

In the range of 1400–400 cm*^−^*^1^ (the fingerprint region), numerous absorption bands are present. Among these, one can identify intense absorptions arising from the deformation vibrations of C-H bonds in alkanes, alkenes, and aromatic hydrocarbons. Additionally, some valence vibrations of C-O single bonds in alcohols, acids, ethers, and esters can also be observed.

### 3.3. Evolution Time of pH and Conductivity in Spirulina platensis Cultures in Different Concentrations of NAs

**Figure 3:**
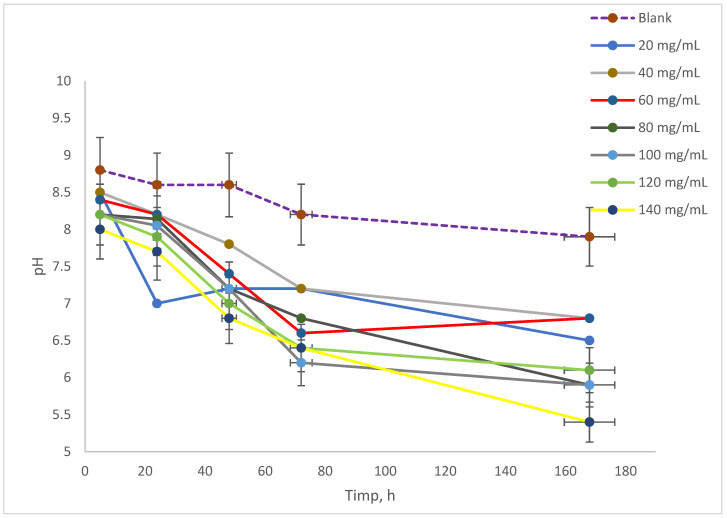
Measurements of pH.

The evolution of pH in the culture medium exposed to varying concentrations of NAs is illustrated in Figure 3. Initially, the pH values of the samples before incubation ranged from 9.5 to 10.0. After 5 h of analysis for series A, it was observed that the pH of the samples contacted with naphthenic acids fell within the range of 8.0 to 8.5, while the blank sample’s pH decreased to 8.8. In terms of the blank sample’s pH progression, it remained stable at 8.6 for up to 48 h, after which it dropped slightly to 8.5 at 72 h, eventually reaching 8.33 by 168 h. The samples that interacted with NAs exhibited a similar pH evolution, with values between 8.0 and 8.5 after 5 h of incubation [15,33]. Over the course of the experiment, the pH continued to decline, ultimately falling within the range of 6.0 to 7.0 by 72 h. By the conclusion of the experiment, all samples displayed lower pH values, reaching a range of 5.5 and below.

The evolution of pH is crucial, as varying concentrations of NAs in the culture of *Spirulina platensis* can impact cell growth. Additionally, monitoring conductivity offers insights into the overall concentration of dissolved salts in the culture medium. Furthermore, tracking oxygen levels is essential, as oxygen plays a key role in metabolic processes within biological environments. This study showed that the disassimilation products of *Spirulina platensis* lead to a decrease in pH. Since the decrease also occurs in the control sample, it is clear that it is not the presence of NA that generates the decrease in pH, but most likely the formation of CO_2_ as a disassimilation product.

**Figure 4:**
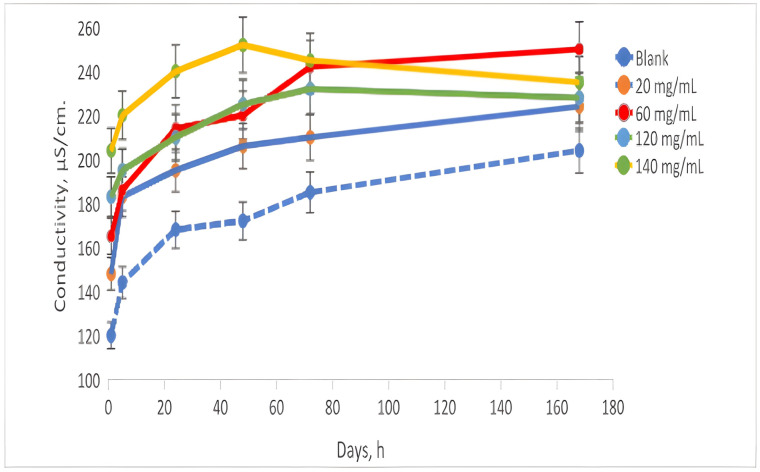
Measurements of conductivity.

The conductivity (Figure 4) of the analyzed samples during the experiment exhibited an initial value ranging from 140 to 200 µS/cm for the samples that were in contact with AN, while the control sample began at 100 µS/cm. Over the course of 72 h, the conductivity values increased; the control sample reached 170 µS/cm, and the contacted samples ranged from 170 to 250 µS/cm. By the conclusion of the experiment, after 168 h, the control sample recorded a conductivity of 190 µS/cm, while the contacted samples remained in the range of 210 to 240 µS/cm.

### 3.4. Quantification of Optical Density (OD) and Sedimentation Index (SI) from Spirulina platensis Cultures

*Spirulina platensis* is a microscopic filamentous cyanobacterium that takes its ‘spiral’ name from the helical nature of its filaments. It is a multicellular filamentous alga, having several spiral shapes. The algal suspension was developed in the specific growth medium and under optimal conditions ensured by continuous agitation in an orbital shaker at 100 rpm, with optimal aeration at 35 °C ± 1 °C. The suspension developed during the experiment was examined microscopically to observe cell viability by measuring the optical density. The values obtained are quantified graphically in Figure 3 following the evolution of the samples contacted with NAs in comparison with the blank sample from the five experimental series. It can be observed that the optical density increases in the blank samples, with the algal suspension reaching an optical density of 0.81 after 168 h of incubation. Initially, the absorbance values of the samples contacted with NAs were between 0.2 and 0.3 after 5 h of incubation, and after 168 h of incubation, they reached different values depending on the concentration of NAs in the samples, in the range between 0.21 and 0.61.

**Figure 5:**
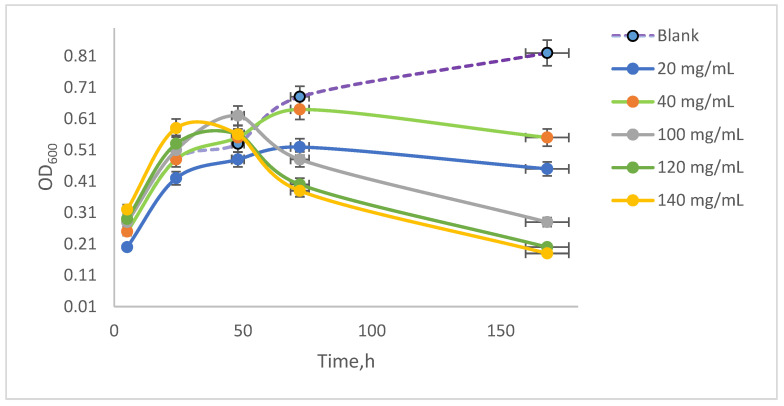
Spectrophotometric measurements of optical density OD_600_.

It was observed that the algal suspension exhibited an increase in optical density (OD_600_) up to 48 h of incubation. Following this period, the samples treated with 140 mg/mL NAs showed a decline in OD, ultimately reaching a value of 0.20 by the end of the experiment. In contrast, the control samples displayed a continuous increase, achieving an OD of 0.81 at the conclusion of the study. The samples treated with 40 mg/mL NAs had an extinction value of 0.60 after 168 h of stirring. These values were illustrated in Figure 5, where the initial extinction measurements were quantified. To assess the differences in extinction due to cellular sedimentation of dead cells that tended to settle, measurements were conducted on the same sample after a 10 min resting period.

**Figure 6:**
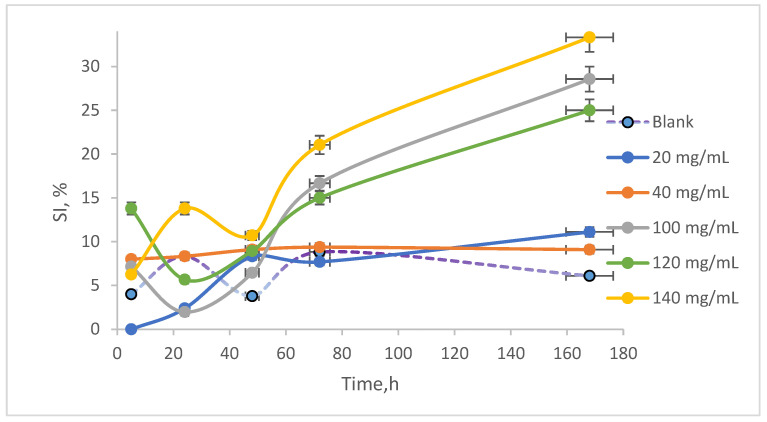
Measurements of SI.

The two measurements allowed for the quantification of the sedimentation index (SI), which is graphically represented in Figure 6. It was observed that the highest SI, approximately 40%, occurred in the sample exposed to 140 mg/mL AN after 168 h of stirring. The control sample exhibited an IS that varied, ultimately reaching around 5% by the end of the experiment. In contrast, the sample treated with 40 mg/mL showed an SI of approximately 6% at the conclusion of the experiment, indicating that this concentration has a minimal impact on cell development and that the chemical stress induced by NAs is manageable.

### 3.5. TEM and Optical Microscopy Visualization of Algae Cells

**Figure 7:**
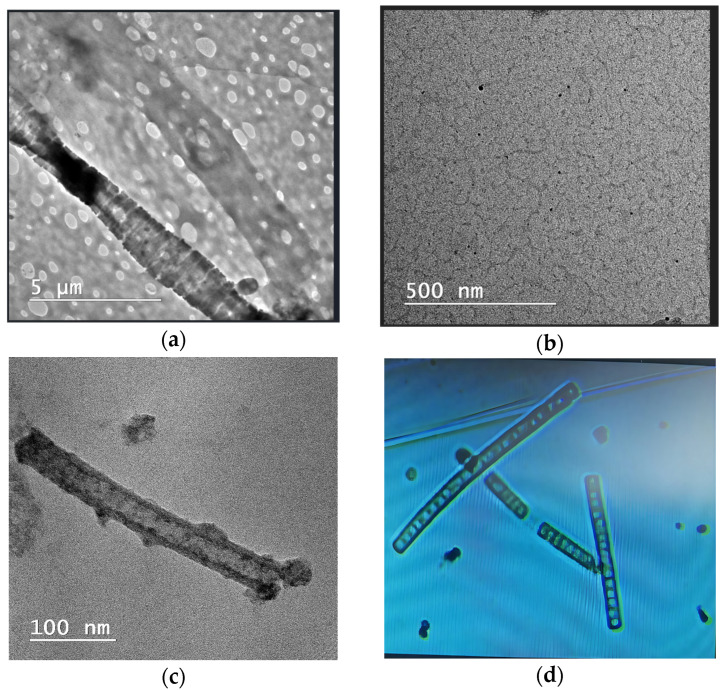
Record of a *Spirulina platensis* from a TEM: surface structure in blank culture algal growth (**a**); strain culture with NAs (140 mg/mL) after 48 h contact (**b**); strain culture with NAs (20 mg/mL) after 168 h contact (**c**); and optical microscopy of blank culture after 24 h, 1200× (**d**).

Microscopic visualization of the samples was conducted following a 10 min resting period, with images obtained through TEM and optical microscopy presented in Figure 7. TEM spectrometry allows the visualization of the structural details of the algal suspension contacted with different concentrations of NA. The images show intact cell walls, proof that the analyzed substance does not cause its destruction. This is also highlighted in Figure 7, where the percentage of growth rate in presence of NAs is presented, quantified as the inhibition ratio (IR). For example, here it is highlighted that for the highest AI concentration tested, 140 mg/mL, the inhibition ratio was 5% after 24 h and 20% after 168 h.

The algae suspension in the blank culture growth (a) can be observed, due to the growing environment in the presence of the NAs at a concentration of 140 mg/mL after 48 h contact and 20 mg/mL after 168 h contact (c). Control biomass of *Spirulina platensis* is detailed with optical microscopy (d).

### 3.6. Chemical Characterization and Confirmation of NAs

**Figure 8:**
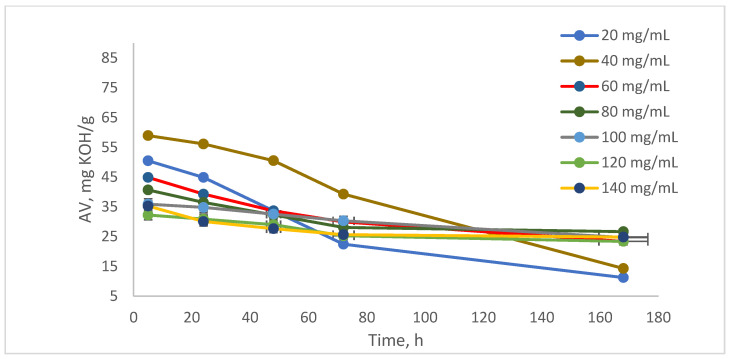
Acidity value analysis.

The analysis of the acidity value (AV) demonstrates that the amount of naphthenic acid (NAs) can be reduced through biological treatment using *Spirulina platensis* (Figure 8). After 5 h of treatment, the acidity value (AV) values ranged from 35 to 85 mg KOH/g of sample. After 72 h, the AV decreased to between 25 and 60 mg KOH/g. Finally, at the end of the test, after 168 h, the AV were recorded at 15 to 35 mg KOH/g of sample [34,35,36].

### 3.7. Inhibition Ratio, Quantification of BOD in the Presence of Nas, and Growth Rate (μ) Evolution

**Figure 9:**
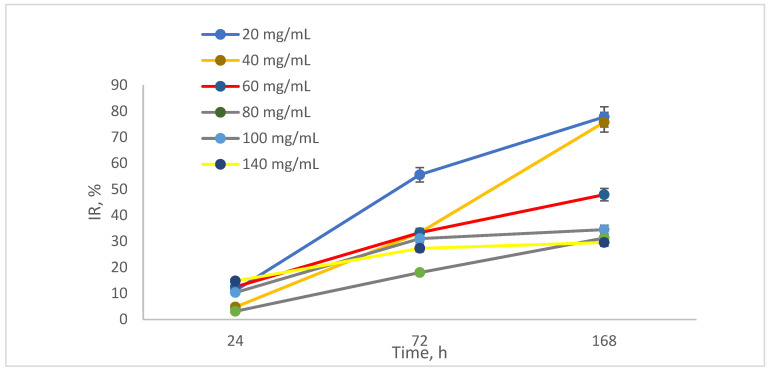
Analysis of the reduction percentage of growth rate in presence of NAs.

The percentage of growth rate in presence of NAs, quantified as the inhibition ratio (IR) is illustrated in Figure 9, demonstrating that after 24 h, the reduction was approximately 13% for the 20 mg/mL sample. After 168 h, the reduction values for the 60 mg/mL and 100 mg/mL samples reached 85%. In terms of inhibition, the percentages were between 5% and 10% after 24 h, increasing to between 25% and 30% after 168 h. For the highest AV concentration tested, 140 mg/mL, the inhibition was 5% after 24 h and 20% after 168 h.

Based on the analysis of experimental values obtained from measuring dissolved oxygen (DO) in the analyzed samples, biological oxygen demand (BOD) was assessed (Figure 8).

**Figure 10:**
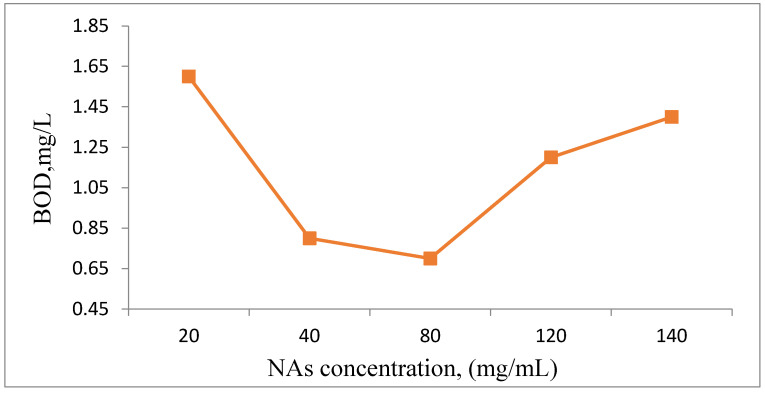
Graphical representation of BOD in the presence of different concentrations of NAs.

It is evident that a significant quantity of NAs can be biodegraded by *Spirulina platensis*. This is attributed to the algae’s cellular metabolic processes, which transform the analyzed substance into metabolic byproducts such as CO_2_ and H_2_O, which are then utilized in respiration. Generally, the presence of inhibitors, such as toxic substances, can lead to cellular inhibition in microorganisms, resulting in a reduction in metabolic processes. Consequently, cellular multiplication may decline; however, cells can often quickly adapt to chemical stressors and develop resistance to inhibitory substances. The detrimental effects of these inhibitors can be lessened by the availability of other substances in the environment that can serve as carbon sources for cellular synthesis. Biological oxygen demand (BOD) indicates that NAs are biodegradable substances that can be utilized in the metabolic processes of *Spirulina platensis* (Figure 10) [35,36,37].

The biodegradation of NAs is influenced by the reactivity and structure of the molecules, as well as their optical configuration. The metabolic transformation of NAs can impact algal cell development over time. One approach to assess the percentage of inhibition involved measuring cell density after a 10 min resting period to quantify the viable algal cells by calculating the growth rate (μ) relative to the concentration of NAs in the sample [37].

**Figure 11:**
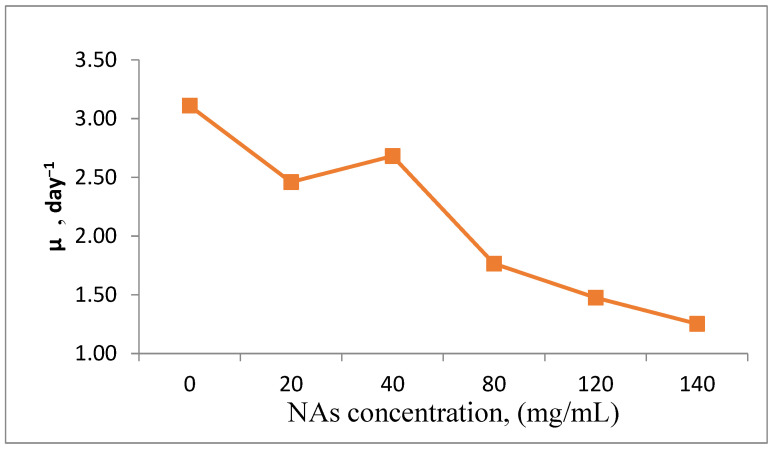
Analysis of growth rate (μ) in the presence of different concentrations of NAs.

From Figure 11, it can be seen that the blank sample had μ = 2.35, day^−1^ compared to the samples contacted with Nas, which had μ 2.50, day^−1^ (the 20 mg/mL sample). During the same time, the 40 mg/mL sample had a higher growth rate, μ 2.75 day^−1^, which means that NAs in this concentration were not inhibitors, but were used as a food source, as a nutritional support for the synthesis of cellular material. A higher concentration of NAs resulted in a lower μ value, between μ 2 and 1.25 day^−1^ for the maximum amount of NAs tested, 140 mg/mL.

### 3.8. Quantification the ECb50 Concentration

**Figure 12:**
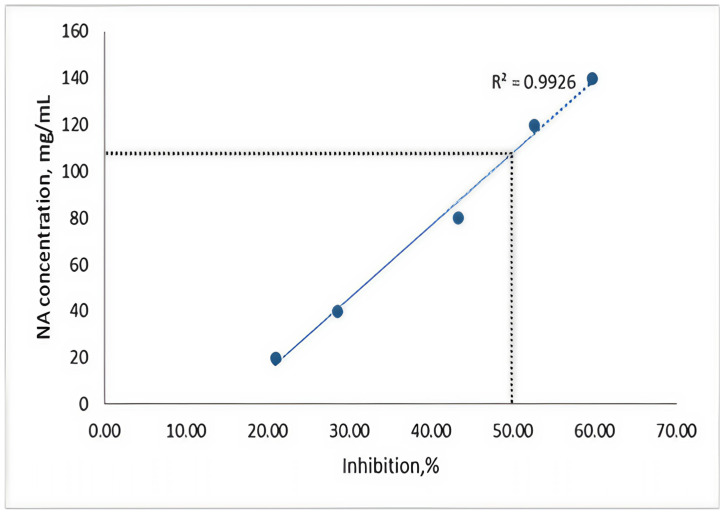
Inhibition percentage of algae cells in the presence of different concentrations of NAs.

Following the tests, we observed the percentage of inhibition that different concentrations of NAs had on algae, highlighting the ECb50 concentration of the substance in relation to algal cell development. The graph depicting the percentage of inhibition (Figure 12) indicates that the analyzed substance inhibits algal growth, thereby affecting photosynthesis. In the initial phase, prior to oxygen consumption in the sample, the toxin exhibits an algistatic effect [36,38,39,40].

However, starting from a concentration of 40 μg/mL, the trend of the curve declines, indicating that NAs has an algicidal effect on *Spirulina platensis*. Despite this, *Spirulina* shows a high tolerance to the pollutant, leading to a low measurement of inhibition based on oxygen production. The curve suggests a moderate toxic action; a concentration of 20 mg/mL results in a 20% inhibition of cell growth, while a concentration of 80 mg/mL induces around 45% growth inhibition. By graphically representing the concentration corresponding to a 50% inhibition, we can estimate the ECb50 value from the regression line, suggesting that a concentration of 110 mg/mL could be inferred. The validity of the results is supported by the slope of the calibration curve, which was calculated with a regression coefficient of R^2^ = 0.9926.

### 3.9. Evaluation of Chemical Changes in NAs and NAD Following Metabolism by Spirulina platensis

**Figure 13:**
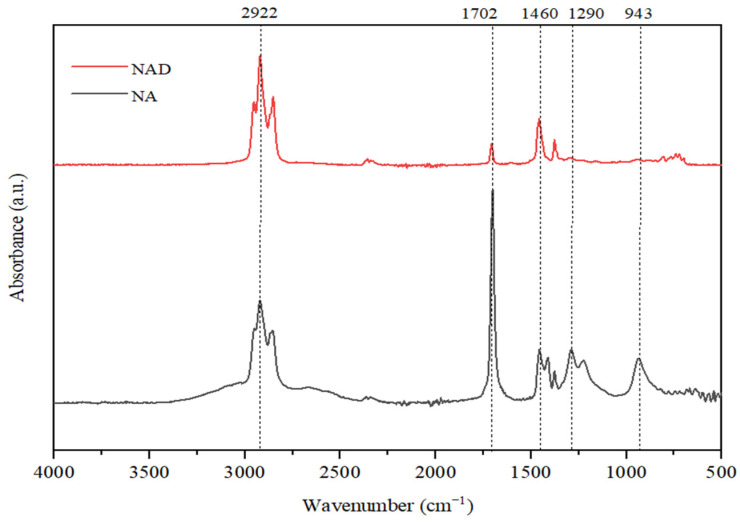
Comparative spectra for biodegraded NAs and NAD samples.

In Figure 13, FTIR analyses of initial NAs and NAD (naphthenic acids distillate) after the microbial metabolism process are presented.

The spectrum analysis reveals that absorption bands at 2922 cm^−1^ can be observed, indicating the presence of valence vibrations of C-H bonds found in alkanes. This peak is more intense for the NAD sample, indicating an increase in the concentration of carbon in paraffinic structures as a result of the degradative processes suffered by naphthenic acids.

A proof of an increased degradation efficiency of naphthenic acids in the presence of *Spirulina platensis* is represented by the absorption band at 1680 cm^−1^, specific to the valence vibrations for the C=O bond in carboxylic structures. For the lower fraction of distilled naphthenic acids, this band is much less intense, which means that naphthenic acids with lower molar mass are much easier to degrade by *Spirulina platensis*. In the NAs sample, this signal is very intense, in line with a high concentration of more complex carboxylic compounds with high molar mass, which are more difficult to degrade.

### 3.10. Influence of the Rate of Biodegradation on the Chemical Composition of NAs

To assess the impact of composition complexity of NAs on degradation efficiency, we separated the lighter fraction from the naphthenic acid sample through high vacuum distillation at a pressure of 0.12 bar and a temperature of 235 °C, and applied the same biodegradation procedure [41].

**Figure 14:**
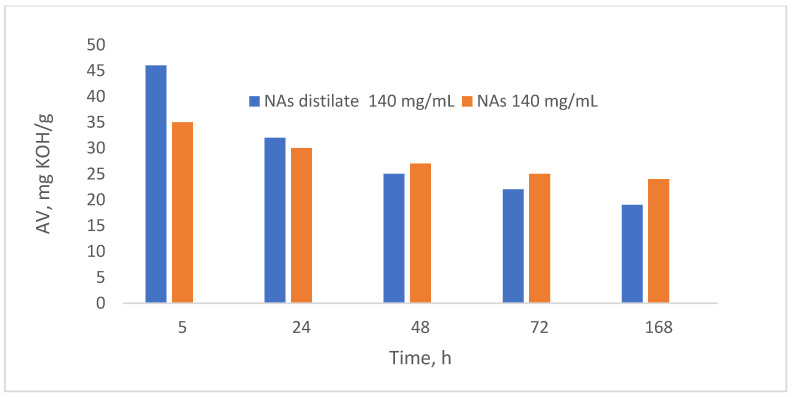
The biodegradation process performances.

Biodegradation was performed at a naphthenic acid concentration of 140 mg/mL. The biodegradation process performances were evaluated by determining the composition of the two biodegradation products. (Figure 14).

The results indicated a higher biodegradation rate compared to the initial naphthenic acid sample, a finding also supported by the literature. For instance, Clemente and Fedorak (2005) [28] noted that naphthenic acids with substituent groups are biodegraded significantly slower than their unsubstituted counterparts [42,43]. Therefore, it follows that naphthenic acids with higher molecular weights, which often include larger substituents, will exhibit slower biodegradation [44,45]. Consequently, lower molecular weight naphthenic acids biodegrade more rapidly, resulting in a higher slope on the acidity value variation curve observed at shorter metabolism times.

The acid value of NAs distillate (140 mg/mL) was initially higher than that of NAs (initial naphthenic acids (140 mg/mL)), but decreased after a steeper slope than that of NAs. This behavior is due to the higher biodegradation rate of NAD than NA. Therefore, increasing the molar mass of NAs decreases the biodegradation rate of NAs.

## 4. Conclusions

The studies conducted highlight the remarkable ability of *Spirulina platensis* algae to biodegrade naphthenic acids. The experiments demonstrate that the algae can be effectively utilized in ecotoxicity tests, owing to their rapid response to toxic stimuli. *Spirulina platensis* is easy to cultivate and exhibits rapid cell growth under laboratory conditions. Analysis of the experimental data reveals that a significant amount of naphthenic acids can be biodegraded by *Spirulina platensis.*

The algae rapidly adapt to chemical stress, developing resistance to inhibitory substances such as naphthenic acids. Tests exposing the *Spirulina platensis* suspension to varying concentrations of naphthenic acids revealed a decrease in pH and an increase in conductivity compared to the initial measurements, attributed to the dissimilation products formed. The acidity index decreased after treatment, suggesting that at the tested concentrations, naphthenic acids did not act as inhibitors. Instead, they appeared to serve as a nutritional resource that supported the synthesis of cellular material. The cell growth rate (μ) at the maximum naphthenic acid concentration of 140 mg/mL was recorded between 2 and 1.25 days^−1^. Additionally, graphical representation of the concentration corresponding to a 50% inhibition allowed for the estimation of the ECb50 value, extrapolated to a naphthenic acid concentration of 110 mg/mL. FTIR analysis of the compositional changes in the naphthenic acid fraction following biodegradation by *Spirulina platensis* indicated a decrease in the concentration of carboxyl groups.

In summary, the tests demonstrate that the analyzed concentrations of naphthenic acids do not significantly impact cell development. The algal suspension is capable of tolerating the toxic chemical stress generated, confirming that naphthenic acids are a class of chemical compounds that *Spirulina platensis* can effectively biodegrade.

## Data Availability

The original contributions presented in this study are included in the article. Further inquiries can be directed to the corresponding author(s).

## Abbreviation

NAs	Naphthenic acids
NAD	Naphthenic acids distillate
OSPW	Oil sands process-affected water
AOPs	Advanced oxidation processes
PAHs	Polycyclic aromatic hydrocarbons
DO	Dissolved oxygen content, mg/L
AV	Acidity value, mg KOH/g
BOD	Biological oxygen demand, mg/L
SI	Sedimentation index, %
OD	Optical density
OD_ti_	Initial optical density measured immediately after harvesting
OD_t10_	Optical density measured after 10 min of rest
IR	Inhibition ratio, %
μ	Average specific growth rate, in days^−1^
ECb50	Concentration of AN corresponding to 50% inhibition, mg/L
Cc	Cell number density of control culture, cells/mL
Ct	Cell number density of samples with specific NA concentrations, cells/mL
FTIR	Fourier transform infrared spectroscopy
